# 7-Bromo-2-phenyl-1-(phenyl­sulfin­yl)naphtho[2,1-*b*]furan benzene hemisolvate

**DOI:** 10.1107/S1600536809025860

**Published:** 2009-07-11

**Authors:** Hong Dae Choi, Pil Ja Seo, Byeng Wha Son, Uk Lee

**Affiliations:** a Department of Chemistry, Dongeui University, San 24 Kaya-dong Busanjin-gu, Busan 614-714, Republic of Korea; b Department of Chemistry Pukyong National University 599-1 Daeyeon 3-dong, Nam-gu, Busan 608-737, Republic of Korea

## Abstract

The title compound, C_24_H_15_BrO_2_S·0.5C_6_H_6_, crystallizes as a benzene hemisolvate. The O atom and the phenyl group of the phenyl­sulfinyl substituent lie on opposite sides of the plane of the naphthofuran fragment, and the phenyl ring is almost perpendicular to the plane of the naphthofuran fragment [83.78 (8)°] and is tilted slightly towards it. The 2-phenyl ring is rotated out of the naphthofuran plane by a dihedral angle of 25.2 (1)°. The crystal structure is stabilized by aromatic π–π inter­actions between the central benzene ring and the furan ring of the neighbouring naphthofuran systems [centroid–centroid distance = 3.611 (3) Å], and by inter­molecular C—H⋯π inter­actions between the benzene H atom of the phenyl­sulfinyl substituent and the 2-phenyl ring of an adjacent mol­ecule. In addition, the crystal structure exhibits a weak non-classical inter­molecular C—H⋯O hydrogen bond.

## Related literature

For the crystal structures of similar 7–bromo­naphtho[2,1–*b*]furan derivatives, see: Choi *et al.* (2007 ▶, 2008 ▶). For details of the biological and pharmacological activity of naphthofuran compounds, see: Goel & Dixit (2004 ▶); Hagiwara *et al.* (1999 ▶); Piloto *et al.* (2005 ▶).
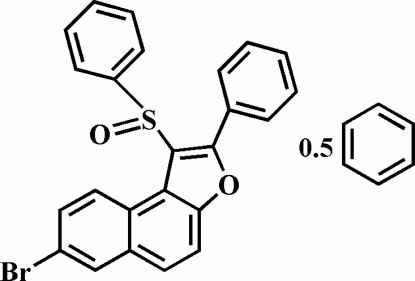


## Experimental

### 

#### Crystal data

C_24_H_15_BrO_2_S·0.5C_6_H_6_
*M* *_r_* = 486.38
Triclinic, 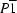

*a* = 8.154 (1) Å
*b* = 10.268 (2) Å
*c* = 13.771 (2) Å
α = 80.656 (2)°
β = 86.431 (2)°
γ = 75.377 (2)°
*V* = 1100.6 (3) Å^3^
*Z* = 2
Mo *K*α radiation
μ = 1.98 mm^−1^
*T* = 298 K
0.40 × 0.10 × 0.10 mm

#### Data collection

Bruker SMART CCD diffractometer
Absorption correction: multi-scan (*SADABS*; Sheldrick, 1999 ▶) *T*_min_ = 0.504, *T*_max_ = 0.826
8221 measured reflections
3870 independent reflections
2968 reflections with *I* > 2σ(*I*)
*R*_int_ = 0.022

#### Refinement

*R*[*F* ^2^ > 2σ(*F* ^2^)] = 0.035
*wR*(*F* ^2^) = 0.091
*S* = 1.06
3870 reflections
280 parameters
3 restraints
H-atom parameters constrained
Δρ_max_ = 0.41 e Å^−3^
Δρ_min_ = −0.74 e Å^−3^

### 

Data collection: *SMART* (Bruker, 2001 ▶); cell refinement: *SAINT* (Bruker, 2001 ▶); data reduction: *SAINT*; program(s) used to solve structure: *SHELXS97* (Sheldrick, 2008 ▶); program(s) used to refine structure: *SHELXL97* (Sheldrick, 2008 ▶); molecular graphics: *ORTEP-3* (Farrugia, 1997 ▶) and *DIAMOND* (Brandenburg, 1998 ▶); software used to prepare material for publication: *SHELXL97*.

## Supplementary Material

Crystal structure: contains datablocks global, I. DOI: 10.1107/S1600536809025860/fl2249sup1.cif

Structure factors: contains datablocks I. DOI: 10.1107/S1600536809025860/fl2249Isup2.hkl

Additional supplementary materials:  crystallographic information; 3D view; checkCIF report

## Figures and Tables

**Table 1:** Hydrogen-bond geometry (Å, °)

*D*—H⋯*A*	*D*—H	H⋯*A*	*D*⋯*A*	*D*—H⋯*A*
C23—H23⋯*Cg*1^i^	0.93	2.87	3.740 (4)	156
C20—H20⋯O2^ii^	0.93	2.52	3.392 (4)	155

Symmetry codes: (i) 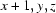; (ii) 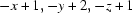. *Cg*1 is the centroid of the C13–C18 benzene ring.

**Fig. 1.**
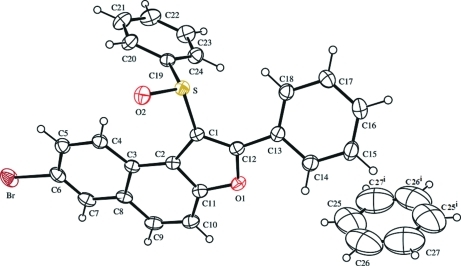
The molecular structure of the title compound with the atom numbering scheme. Displacement ellipsoids are drawn at the 30% probability level. H atoms are presented as a small spheres of arbitrary radius. [Symmetry code: (i) -x+1, -y, -z+2.]

**Fig. 2.**
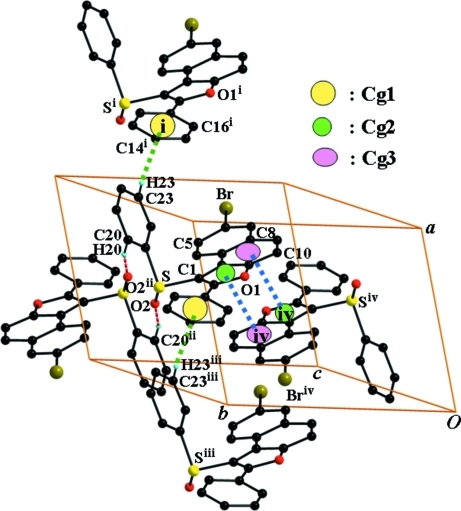
The π–π, C—H···π and C—H···O interactions (dotted lines) in the title compound. Cg denotes the ring centroids. [Symmetry codes: (i) x+1, y, z; (ii) -x+1, -y+2, -z+1; (iii) x-1, y, z; (iv) -x+1, -y+1, -z+1.]

## supplementary crystallographic information

### Comment

Naphthofuran compounds have attracted widespread interest in view of their biological and pharmacological activities (Goel & Dixit, 2004; Hagiwara *et al.*, 1999; Piloto *et al.*, 2005). This work is related to our communications on the synthesis and structures of 7–bromonaphtho[2,1–*b*]furan analogues, *viz*. 7–bromo–2–methyl–1–(phenylsulfanyl)naphtho[2,1–*b*]furan (Choi *et al.*, 2007) and 7–bromo–2–methyl–1–(phenylsulfonyl)naphtho[2,1–*b*]furan (Choi *et al.*, 2008). We present here the crystal structure of the title compound (I) co-crystallized with benzene. The benzene solvate sits on a center of symmetry (Fig. 1) such that the structures is a hemisolvate.

The naphthofuran unit is essentially planar, with a mean deviation of 0.056 (2) Å from the least–squares plane defined by the thirteen constituent atoms. The dihedral angle in (I) formed by the plane of the naphthofuran ring and the plane of 2–phenyl ring is 25.2 (1)°, and the phenyl ring (C19–C24) with a dihedral angle of 83.78 (8)° is almost perpendicular and titlled slightly toward the naphthofuran moieity. The crystal packing (Fig. 2) is stabilized by aromatic π–π interactions between the central benzene ring and the furan ring from  adjacent molecules. The Cg2···Cg3^iv^ distance is 3.611 (3) Å (Cg2 and Cg3 are the centroides of the C1/C2/C11/O1/C12 furan ring and the C2/C3/C8/C9/C10/C11 benzene ring, respectively, symmetry code as in Fig. 2). The molecular packing is further stabilized by intermolecular C—H···π interactions between the benzene H atom of the phenylsulfinyl substituent and the 2-phenyl ring of an adjacent molecule (Table 1 and Fig. 2). Additionally, a weak non–calssical intermolecular C—H···O hydrogen bond in the structure was observed (Fig. 2 and Table 1; symmetry code as in Fig. 2).

### Experimental

3-chloroperoxybenzoic acid (77%, 217 mg, 0.88 mmol) was added in small portions to a stirred solution of 7-bromo-2-phenyl-1-(phenylsulfanyl)naphtho[2,1–*b*]furan (345 mg, 0.8 mmol) in dichloromethane (40 mL) at 273 K. After being stirred at room temperature for 4h, the mixture was washed with saturated sodium bicarbonate solution and the organic layer was separated, dried over magnesium sulfate, filtered and concentrated in vacuum. The residue was purified by column chromatography (hexane-ethyl acetate, 2:1 v/v) to afford the title compound as a colorless solid [yield 78%, m.p. 448–449 K; *R*_f_ = 0.60 (hexane-ethyl acetate, 2:1 v/v)]. Single crystals suitable for X–ray diffraction were prepared by slow evaporation of a solution of the title compound in benzene at room temperature.

### Refinement

All H atoms were positioned geometrically and refined using a riding model, with C—H = 0.93 Å for aromatic H atoms and with Uiso(H) = 1.2Ueq(C) for aromatic H atoms. The distances of C—C in the solvated benzene ring were restrained to 1.40 (1) Å using command DFIX.

### Crystal data

**Table:**

C_24_H_15_BrO_2_S·0.5C_6_H_6_	*Z* = 2
*M**_r_* = 486.38	*F*(000) = 494
Triclinic, *P*1	*D*_x_ = 1.468 Mg m^−^^3^
Hall symbol: -p 1	Mo *K*α radiation, λ = 0.71073 Å
*a* = 8.154 (1) Å	Cell parameters from 3339 reflections
*b* = 10.268 (2) Å	θ = 2.4–26.4°
*c* = 13.771 (2) Å	µ = 1.98 mm^−^^1^
α = 80.656 (2)°	*T* = 298 K
β = 86.431 (2)°	Block, colorless
γ = 75.377 (2)°	0.40 × 0.10 × 0.10 mm
*V* = 1100.6 (3) Å^3^	

### Data collection

**Table:**

Bruker SMART CCD diffractometer	3870 independent reflections
Radiation source: fine-focus sealed tube	2968 reflections with *I* > 2σ(*I*)
graphite	*R*_int_ = 0.022
Detector resolution: 10.0 pixels mm^-1^	θ_max_ = 25.0°, θ_min_ = 1.5°
φ and ω scans	*h* = −9→9
Absorption correction: multi-scan (*SADABS*; Sheldrick, 1999)	*k* = −12→12
*T*_min_ = 0.504, *T*_max_ = 0.826	*l* = −16→16
8221 measured reflections	

### Refinement

**Table:**

Refinement on *F*^2^	Primary atom site location: structure-invariant direct methods
Least-squares matrix: full	Secondary atom site location: difference Fourier map
*R*[*F*^2^ > 2σ(*F*^2^)] = 0.035	Hydrogen site location: difference Fourier map
*wR*(*F*^2^) = 0.091	H-atom parameters constrained
*S* = 1.06	*w* = 1/[σ^2^(*F*_o_^2^) + (0.0375*P*)^2^ + 0.6613*P*] where *P* = (*F*_o_^2^ + 2*F*_c_^2^)/3
3870 reflections	(Δ/σ)_max_ < 0.001
280 parameters	Δρ_max_ = 0.41 e Å^−^^3^
3 restraints	Δρ_min_ = −0.73 e Å^−^^3^

### Special details

**Table:**

Geometry. All esds (except the esd in the dihedral angle between two l.s. planes) are estimated using the full covariance matrix. The cell esds are taken into account individually in the estimation of esds in distances, angles and torsion angles; correlations between esds in cell parameters are only used when they are defined by crystal symmetry. An approximate (isotropic) treatment of cell esds is used for estimating esds involving l.s. planes.
Refinement. Refinement of F^2^ against ALL reflections. The weighted R-factor wR and goodness of fit S are based on F^2^, conventional R-factors R are based on F, with F set to zero for negative F^2^. The threshold expression of F^2^ > 2sigma(F^2^) is used only for calculating R-factors(gt) etc. and is not relevant to the choice of reflections for refinement. R-factors based on F^2^ are statistically about twice as large as those based on F, and R- factors based on ALL data will be even larger.

### Fractional atomic coordinates and isotropic or equivalent isotropic displacement parameters (Å^2^)

**Table:**

	*x*	*y*	*z*	*U*_iso_*/*U*_eq_	
Br	1.02842 (5)	0.72284 (4)	0.21176 (3)	0.06839 (16)	
S	0.47922 (8)	0.81504 (7)	0.68119 (5)	0.03579 (18)	
O1	0.5475 (2)	0.41820 (18)	0.70208 (14)	0.0414 (5)	
O2	0.4093 (2)	0.90091 (19)	0.58833 (15)	0.0458 (5)	
C1	0.5365 (3)	0.6416 (3)	0.66622 (19)	0.0329 (6)	
C2	0.6310 (3)	0.5754 (3)	0.58705 (19)	0.0338 (6)	
C3	0.7147 (3)	0.6160 (3)	0.4973 (2)	0.0351 (6)	
C4	0.7098 (4)	0.7522 (3)	0.4566 (2)	0.0402 (7)	
H4	0.6442	0.8226	0.4874	0.048*	
C5	0.8001 (4)	0.7824 (3)	0.3723 (2)	0.0440 (7)	
H5	0.7966	0.8725	0.3465	0.053*	
C6	0.8974 (4)	0.6769 (4)	0.3254 (2)	0.0459 (7)	
C7	0.8991 (4)	0.5447 (3)	0.3587 (2)	0.0475 (8)	
H7	0.9617	0.4767	0.3246	0.057*	
C8	0.8064 (3)	0.5098 (3)	0.4450 (2)	0.0395 (7)	
C9	0.8021 (4)	0.3721 (3)	0.4790 (2)	0.0486 (8)	
H9	0.8611	0.3048	0.4432	0.058*	
C10	0.7148 (4)	0.3359 (3)	0.5616 (2)	0.0475 (7)	
H10	0.7102	0.2460	0.5823	0.057*	
C11	0.6323 (3)	0.4400 (3)	0.6141 (2)	0.0386 (6)	
C12	0.4902 (3)	0.5427 (3)	0.7334 (2)	0.0362 (6)	
C13	0.3985 (3)	0.5386 (3)	0.8286 (2)	0.0369 (6)	
C14	0.3248 (4)	0.4299 (3)	0.8606 (2)	0.0483 (8)	
H14	0.3349	0.3620	0.8218	0.058*	
C15	0.2368 (4)	0.4225 (4)	0.9498 (2)	0.0558 (9)	
H15	0.1892	0.3491	0.9708	0.067*	
C16	0.2190 (4)	0.5227 (4)	1.0078 (2)	0.0545 (8)	
H16	0.1571	0.5184	1.0668	0.065*	
C17	0.2934 (4)	0.6293 (3)	0.9780 (2)	0.0487 (8)	
H17	0.2833	0.6964	1.0176	0.058*	
C18	0.3836 (4)	0.6372 (3)	0.8893 (2)	0.0438 (7)	
H18	0.4345	0.7091	0.8702	0.053*	
C19	0.6897 (3)	0.8360 (3)	0.6940 (2)	0.0360 (6)	
C20	0.7429 (4)	0.9400 (3)	0.6352 (2)	0.0482 (8)	
H20	0.6740	0.9970	0.5864	0.058*	
C21	0.9020 (4)	0.9576 (4)	0.6506 (3)	0.0625 (9)	
H21	0.9417	1.0254	0.6101	0.075*	
C22	1.0016 (4)	0.8762 (4)	0.7248 (3)	0.0636 (10)	
H22	1.1070	0.8903	0.7353	0.076*	
C23	0.9455 (4)	0.7741 (4)	0.7834 (3)	0.0593 (9)	
H23	1.0130	0.7191	0.8337	0.071*	
C24	0.7895 (4)	0.7527 (3)	0.7681 (2)	0.0476 (7)	
H24	0.7519	0.6829	0.8074	0.057*	
C25	0.599 (4)	0.0294 (11)	0.9196 (17)	0.195 (5)	
H25	0.6656	0.0503	0.8648	0.233*	
C26	0.432 (5)	0.0346 (11)	0.9090 (14)	0.191 (5)	
H26	0.3862	0.0575	0.8466	0.229*	
C27	0.3318 (17)	0.0065 (14)	0.989 (3)	0.204 (5)	
H27	0.2177	0.0118	0.9810	0.245*	

### Atomic displacement parameters (Å^2^)

**Table:**

	*U*^11^	*U*^22^	*U*^33^	*U*^12^	*U*^13^	*U*^23^
Br	0.0633 (2)	0.1022 (3)	0.0468 (2)	−0.0310 (2)	0.01776 (16)	−0.02234 (19)
S	0.0340 (4)	0.0318 (4)	0.0395 (4)	−0.0043 (3)	0.0015 (3)	−0.0064 (3)
O1	0.0450 (11)	0.0316 (10)	0.0488 (12)	−0.0119 (9)	0.0004 (9)	−0.0058 (9)
O2	0.0412 (11)	0.0394 (11)	0.0493 (12)	−0.0007 (9)	−0.0051 (9)	0.0019 (9)
C1	0.0308 (14)	0.0307 (14)	0.0374 (15)	−0.0069 (11)	−0.0039 (11)	−0.0050 (12)
C2	0.0304 (14)	0.0321 (15)	0.0382 (15)	−0.0040 (11)	−0.0042 (11)	−0.0078 (12)
C3	0.0296 (14)	0.0386 (16)	0.0379 (15)	−0.0054 (12)	−0.0032 (11)	−0.0122 (13)
C4	0.0390 (15)	0.0408 (17)	0.0406 (16)	−0.0072 (13)	0.0020 (12)	−0.0109 (13)
C5	0.0438 (16)	0.0482 (18)	0.0411 (17)	−0.0133 (14)	0.0013 (13)	−0.0081 (14)
C6	0.0370 (16)	0.067 (2)	0.0376 (16)	−0.0148 (15)	0.0028 (13)	−0.0161 (15)
C7	0.0379 (16)	0.061 (2)	0.0458 (18)	−0.0050 (15)	−0.0007 (13)	−0.0255 (16)
C8	0.0324 (14)	0.0437 (17)	0.0432 (16)	−0.0031 (13)	−0.0054 (12)	−0.0172 (14)
C9	0.0453 (17)	0.0418 (18)	0.059 (2)	−0.0006 (14)	−0.0038 (15)	−0.0248 (15)
C10	0.0506 (18)	0.0345 (17)	0.058 (2)	−0.0077 (14)	−0.0061 (15)	−0.0115 (15)
C11	0.0372 (15)	0.0357 (16)	0.0438 (16)	−0.0082 (13)	−0.0051 (13)	−0.0081 (13)
C12	0.0324 (14)	0.0357 (16)	0.0409 (16)	−0.0086 (12)	−0.0059 (12)	−0.0050 (13)
C13	0.0325 (14)	0.0393 (16)	0.0380 (16)	−0.0101 (12)	−0.0064 (12)	0.0008 (13)
C14	0.0516 (18)	0.0526 (19)	0.0446 (18)	−0.0212 (15)	−0.0074 (14)	−0.0030 (15)
C15	0.058 (2)	0.062 (2)	0.0495 (19)	−0.0296 (17)	0.0002 (16)	0.0068 (17)
C16	0.0504 (19)	0.070 (2)	0.0393 (17)	−0.0151 (17)	0.0028 (14)	0.0013 (17)
C17	0.0518 (18)	0.0525 (19)	0.0388 (17)	−0.0080 (15)	−0.0019 (14)	−0.0061 (14)
C18	0.0456 (17)	0.0441 (17)	0.0413 (17)	−0.0136 (14)	−0.0035 (13)	−0.0004 (14)
C19	0.0354 (14)	0.0325 (15)	0.0400 (16)	−0.0052 (12)	−0.0016 (12)	−0.0097 (12)
C20	0.0523 (19)	0.0350 (17)	0.058 (2)	−0.0147 (14)	−0.0078 (15)	0.0010 (14)
C21	0.059 (2)	0.050 (2)	0.084 (3)	−0.0277 (18)	0.0003 (19)	−0.0043 (19)
C22	0.0402 (18)	0.060 (2)	0.096 (3)	−0.0156 (17)	−0.0098 (19)	−0.018 (2)
C23	0.0481 (19)	0.058 (2)	0.069 (2)	−0.0060 (17)	−0.0187 (17)	−0.0054 (18)
C24	0.0461 (18)	0.0440 (18)	0.0513 (19)	−0.0105 (15)	−0.0059 (14)	−0.0023 (15)
C25	0.28 (2)	0.110 (6)	0.176 (15)	0.006 (9)	0.013 (10)	−0.058 (7)
C26	0.268 (19)	0.109 (6)	0.179 (15)	0.019 (10)	−0.068 (14)	−0.052 (8)
C27	0.211 (11)	0.117 (7)	0.281 (16)	0.002 (8)	−0.05 (2)	−0.074 (10)

### Geometric parameters (Å, °)

**Table:**

Br—C6	1.902 (3)	C14—C15	1.385 (4)
S—O2	1.486 (2)	C14—H14	0.9300
S—C1	1.767 (3)	C15—C16	1.376 (5)
S—C19	1.805 (3)	C15—H15	0.9300
O1—C11	1.371 (3)	C16—C17	1.375 (4)
O1—C12	1.375 (3)	C16—H16	0.9300
C1—C12	1.369 (4)	C17—C18	1.388 (4)
C1—C2	1.455 (4)	C17—H17	0.9300
C2—C11	1.377 (4)	C18—H18	0.9300
C2—C3	1.428 (4)	C19—C20	1.376 (4)
C3—C4	1.411 (4)	C19—C24	1.379 (4)
C3—C8	1.429 (4)	C20—C21	1.387 (4)
C4—C5	1.370 (4)	C20—H20	0.9300
C4—H4	0.9300	C21—C22	1.373 (5)
C5—C6	1.396 (4)	C21—H21	0.9300
C5—H5	0.9300	C22—C23	1.371 (5)
C6—C7	1.357 (4)	C22—H22	0.9300
C7—C8	1.414 (4)	C23—C24	1.379 (4)
C7—H7	0.9300	C23—H23	0.9300
C8—C9	1.423 (4)	C24—H24	0.9300
C9—C10	1.354 (4)	C25—C26	1.37 (5)
C9—H9	0.9300	C25—C27^i^	1.37 (4)
C10—C11	1.396 (4)	C25—H25	0.9300
C10—H10	0.9300	C26—C27	1.37 (4)
C12—C13	1.468 (4)	C26—H26	0.9300
C13—C18	1.391 (4)	C27—C25^i^	1.37 (4)
C13—C14	1.396 (4)	C27—H27	0.9300
			
O2—S—C1	110.10 (12)	C15—C14—C13	120.4 (3)
O2—S—C19	107.93 (12)	C15—C14—H14	119.8
C1—S—C19	97.94 (12)	C13—C14—H14	119.8
C11—O1—C12	106.8 (2)	C16—C15—C14	120.7 (3)
C12—C1—C2	107.4 (2)	C16—C15—H15	119.7
C12—C1—S	122.2 (2)	C14—C15—H15	119.7
C2—C1—S	130.4 (2)	C17—C16—C15	119.6 (3)
C11—C2—C3	119.0 (2)	C17—C16—H16	120.2
C11—C2—C1	104.3 (2)	C15—C16—H16	120.2
C3—C2—C1	136.7 (2)	C16—C17—C18	120.3 (3)
C4—C3—C2	124.6 (2)	C16—C17—H17	119.8
C4—C3—C8	118.6 (3)	C18—C17—H17	119.8
C2—C3—C8	116.7 (3)	C17—C18—C13	120.7 (3)
C5—C4—C3	121.0 (3)	C17—C18—H18	119.7
C5—C4—H4	119.5	C13—C18—H18	119.7
C3—C4—H4	119.5	C20—C19—C24	121.2 (3)
C4—C5—C6	119.6 (3)	C20—C19—S	119.9 (2)
C4—C5—H5	120.2	C24—C19—S	118.7 (2)
C6—C5—H5	120.2	C19—C20—C21	118.4 (3)
C7—C6—C5	121.6 (3)	C19—C20—H20	120.8
C7—C6—Br	120.2 (2)	C21—C20—H20	120.8
C5—C6—Br	118.2 (2)	C22—C21—C20	120.8 (3)
C6—C7—C8	120.4 (3)	C22—C21—H21	119.6
C6—C7—H7	119.8	C20—C21—H21	119.6
C8—C7—H7	119.8	C23—C22—C21	119.9 (3)
C7—C8—C9	121.1 (3)	C23—C22—H22	120.0
C7—C8—C3	118.6 (3)	C21—C22—H22	120.0
C9—C8—C3	120.3 (3)	C22—C23—C24	120.3 (3)
C10—C9—C8	122.2 (3)	C22—C23—H23	119.8
C10—C9—H9	118.9	C24—C23—H23	119.8
C8—C9—H9	118.9	C23—C24—C19	119.3 (3)
C9—C10—C11	116.7 (3)	C23—C24—H24	120.3
C9—C10—H10	121.7	C19—C24—H24	120.3
C11—C10—H10	121.7	C26—C25—C27^i^	119.8 (11)
O1—C11—C2	111.6 (2)	C26—C25—H25	120.1
O1—C11—C10	123.5 (3)	C27^i^—C25—H25	120.1
C2—C11—C10	124.9 (3)	C25—C26—C27	120.9 (12)
C1—C12—O1	109.9 (2)	C25—C26—H26	119.5
C1—C12—C13	135.7 (3)	C27—C26—H26	119.5
O1—C12—C13	114.5 (2)	C26—C27—C25^i^	119.3 (11)
C18—C13—C14	118.3 (3)	C26—C27—H27	120.4
C18—C13—C12	123.1 (3)	C25^i^—C27—H27	120.4
C14—C13—C12	118.6 (3)		
			
O2—S—C1—C12	−132.3 (2)	C9—C10—C11—O1	−176.0 (3)
C19—S—C1—C12	115.2 (2)	C9—C10—C11—C2	1.2 (4)
O2—S—C1—C2	46.3 (3)	C2—C1—C12—O1	−0.6 (3)
C19—S—C1—C2	−66.2 (3)	S—C1—C12—O1	178.36 (17)
C12—C1—C2—C11	0.4 (3)	C2—C1—C12—C13	178.5 (3)
S—C1—C2—C11	−178.4 (2)	S—C1—C12—C13	−2.6 (4)
C12—C1—C2—C3	−179.2 (3)	C11—O1—C12—C1	0.5 (3)
S—C1—C2—C3	2.0 (5)	C11—O1—C12—C13	−178.8 (2)
C11—C2—C3—C4	173.8 (3)	C1—C12—C13—C18	−22.7 (5)
C1—C2—C3—C4	−6.6 (5)	O1—C12—C13—C18	156.3 (3)
C11—C2—C3—C8	−4.8 (4)	C1—C12—C13—C14	158.2 (3)
C1—C2—C3—C8	174.8 (3)	O1—C12—C13—C14	−22.7 (3)
C2—C3—C4—C5	177.0 (3)	C18—C13—C14—C15	1.1 (4)
C8—C3—C4—C5	−4.4 (4)	C12—C13—C14—C15	−179.8 (3)
C3—C4—C5—C6	0.6 (4)	C13—C14—C15—C16	0.7 (5)
C4—C5—C6—C7	3.0 (4)	C14—C15—C16—C17	−1.8 (5)
C4—C5—C6—Br	−177.4 (2)	C15—C16—C17—C18	1.1 (5)
C5—C6—C7—C8	−2.4 (4)	C16—C17—C18—C13	0.7 (5)
Br—C6—C7—C8	177.9 (2)	C14—C13—C18—C17	−1.8 (4)
C6—C7—C8—C9	177.7 (3)	C12—C13—C18—C17	179.1 (3)
C6—C7—C8—C3	−1.5 (4)	O2—S—C19—C20	13.1 (3)
C4—C3—C8—C7	4.9 (4)	C1—S—C19—C20	127.3 (2)
C2—C3—C8—C7	−176.4 (2)	O2—S—C19—C24	−171.6 (2)
C4—C3—C8—C9	−174.4 (3)	C1—S—C19—C24	−57.4 (2)
C2—C3—C8—C9	4.3 (4)	C24—C19—C20—C21	1.5 (5)
C7—C8—C9—C10	179.7 (3)	S—C19—C20—C21	176.7 (2)
C3—C8—C9—C10	−1.0 (4)	C19—C20—C21—C22	−2.2 (5)
C8—C9—C10—C11	−1.7 (4)	C20—C21—C22—C23	1.4 (6)
C12—O1—C11—C2	−0.3 (3)	C21—C22—C23—C24	0.0 (6)
C12—O1—C11—C10	177.2 (3)	C22—C23—C24—C19	−0.7 (5)
C3—C2—C11—O1	179.7 (2)	C20—C19—C24—C23	0.0 (5)
C1—C2—C11—O1	0.0 (3)	S—C19—C24—C23	−175.3 (2)
C3—C2—C11—C10	2.2 (4)	C27^i^—C25—C26—C27	1.1 (19)
C1—C2—C11—C10	−177.5 (3)	C25—C26—C27—C25^i^	−1.1 (19)

Symmetry codes: (i) −*x*+1, −*y*, −*z*+2.

### Hydrogen-bond geometry (Å, °)

**Table:**

*D*—H···*A*	*D*—H	H···*A*	*D*···*A*	*D*—H···*A*
C23—H23···Cg1^ii^	0.93	2.87	3.740 (4)	156
C20—H20···O2^iii^	0.93	2.52	3.392 (4)	155

Symmetry codes: (ii) *x*+1, *y*, *z*; (iii) −*x*+1, −*y*+2, −*z*+1.

## References

[bb1] Brandenburg, K. (1998). *DIAMOND* Crystal Impact GbR, Bonn, Germany.

[bb2] Bruker (2001). *SAINT* and *SMART* Bruker AXS Inc., Madison, Wisconsin, USA.

[bb3] Choi, H. D., Seo, P. J., Son, B. W. & Lee, U. (2007). *Acta Cryst.* E**63**, o4102.

[bb4] Choi, H. D., Seo, P. J., Son, B. W. & Lee, U. (2008). *Acta Cryst.* E**64**, o944. 10.1107/S160053680801177X PMC2961282 21202425

[bb5] Farrugia, L. J. (1997). *J. Appl. Cryst.* **30**, 565.

[bb6] Goel, A. & Dixit, M. (2004). *Tetrahedron Lett* **45**, 8819–8821.

[bb7] Hagiwara, H., Sato, K., Suzuki, T. & Ando, M. (1999). *Heterocycles*, **51**, 497–500.

[bb8] Piloto, A. M., Costa, S. P. G. & Goncalves, M. S. T. (2005). *Tetrahedron Lett* **46**, 4757–4760.

[bb9] Sheldrick, G. M. (1999). *SADABS* University of Göttingen, Germany.

[bb10] Sheldrick, G. M. (2008). *Acta Cryst.* A**64**, 112–122. 10.1107/S0108767307043930 18156677

